# Communicating Risk to Aboriginal Peoples: First Nations and Metis Responses to H1N1 Risk Messages

**DOI:** 10.1371/journal.pone.0071106

**Published:** 2013-08-07

**Authors:** S. Michelle Driedger, Elizabeth Cooper, Cindy Jardine, Chris Furgal, Judith Bartlett

**Affiliations:** 1 Department of Community Health Sciences, Faculty of Medicine, University of Manitoba, Winnipeg, Manitoba, Canada; 2 School of Public Health, University of Alberta, Edmonton, Alberta, Canada; 3 Indigenous Environment Studies Program, Trent University, Peterborough, Ontario, Canada; Melbourne School of Population Health, Australia

## Abstract

Developing appropriate risk messages during challenging situations like public health outbreaks is complicated. The focus of this paper is on how First Nations and Metis people in Manitoba, Canada, responded to the public health management of pandemic H1N1, using a focus group methodology (n = 23 focus groups). Focus group conversations explored participant reactions to messaging regarding the identification of H1N1 virus risk groups, the H1N1 vaccine and how priority groups to receive the vaccine were established. To better contextualize the intentions of public health professionals, key informant interviews (n = 20) were conducted with different health decision makers (e.g., public health officials, people responsible for communications, representatives from some First Nations and Metis self-governing organizations). While risk communication practice has improved, ‘one size’ messaging campaigns do not work effectively, particularly when communicating about who is most ‘at-risk’. Public health agencies need to pay more attention to the specific socio-economic, historical and cultural contexts of First Nations and Metis citizens when planning for, communicating and managing responses associated with pandemic outbreaks to better tailor both the messages and delivery. More attention is needed to directly engage First Nations and Metis communities in the development and dissemination of risk messaging.

## Introduction

Pandemic H1N1, the first pandemic of the 21^st^ century, had constant media reporting. Internet and social media sites were active with available, but not always credible, information and globally, countries were responding to the pandemic differently. The public health system response to pandemic H1N1 provided many countries, including Canada, an opportunity for its multiple jurisdictions to identify what worked well (or not) from its pandemic planning documents and preparedness activities, including the development of public risk communication messaging, so that lessons could be learned for future improvements.

The research presented in this article is taken from the results of one national research study undertaken to examine the effectiveness of public health risk communication messaging with Manitoba Metis [1], [2]. Data from the study examined here is drawn from focus groups and key informant interviews. The focus groups examined how Manitoba First Nations and Metis understood messaging about pandemic H1N1 influenza risk and protective measures, including the designation of Aboriginal people as a priority for receiving the vaccine. Key informant interviews examining the intentions and actions of health decision-makers in the management of pandemic H1N1 were conducted with public health and communications officials and representatives from some First Nations and Metis self-governing organizations.

## Background

### Aboriginal People in Canada and Pandemic H1N1

In Canada, ‘Aboriginal’ is constitutionally defined as Indian (hereafter referred to as First Nations unless specific to government legislation), Inuit and Métis peoples [3]. First Nations refers to Aboriginal peoples who are neither Inuit (i.e., Arctic-settled people thought to be descendants of the Thule culture) nor Metis [4]. Metis refers to people of mixed First Nations-European heritage (Manitoba is viewed as the ‘birthplace’ of the Metis nation, and the Manitoba Metis Federation, the democratic self-governing political representative of the Manitoba Metis Nation, does not use the accented “Métis” in their writing. For these reasons, the preferred representation of the unaccented Metis is adopted in this article) [5], [6]. Generally speaking, the federal government is fiscally responsible for health and social service delivery for First Nations (i.e. those who are registered under the Indian Act of Canada [7]), whereas Metis citizens fall under the primary jurisdiction of the province or territory in which they live [8], [9]. Some of this may change given recent court decisions [10], [11]. Regardless, Aboriginal people have had a strong negative history concerning their health and accessing services in Canada [12], [13], [14], [15]. How individuals are identified as ‘Aboriginal’ is complex, resulting in difficulty in how rates of H1N1 in Aboriginal people are represented in the literature. Numbers of H1N1 in Aboriginal people reported in Canada [16], [17] and Manitoba [18] are ostensibly based on individuals self-identifying as registered First Nations, Inuit, or Metis. Given H1N1 case reports in Manitoba First Nations people during pandemic Wave 1 [16] and the acknowledged difficulty linking H1N1 cases with Manitoba Metis citizens, rates of H1N1 in Aboriginal people in Manitoba are largely assumed to be representative of people of First Nations ancestry (Manitoba Metis Federation Key Informant Interview Data).

In Canada, approximately 8,700 hospitalizations and 428 deaths were estimated as a result of the entire H1N1 pandemic (∼April 2009–August 2010), of which 10% of each were Aboriginal people - a particularly dire outcome given that Aboriginal people represent approximately 4% of the Canadian population [19]. In Wave 1 (∼ April–August 2009), people of Aboriginal descent constituted 46% of hospitalizations and 18% of deaths, mainly in the province of Manitoba. This dropped to 6% of hospitalizations and 9% of deaths for Aboriginal people in Wave 2 (∼ October 2009–August 2010), mostly among Aboriginal people in the province of Alberta [16]. Of the total number of critically ill patients treated in Canadian hospitals during Wave 1, 25% were Aboriginal [17].

Pandemic H1N1 hit Aboriginal populations in Canada disproportionately hard, pointing to a broader history of poorer health outcomes for Aboriginal people. When compared to general-population Canadians, the health status of Aboriginal people is consistently lower. The current state of Aboriginal health, and its potential vulnerability during a pandemic, is increasingly recognized as the result of complex political and socio-economic factors and long colonial histories existing in local and national contexts [20]. Similar findings have been identified for other Aboriginal communities internationally [21], [22].

### Public Health Pandemic Risk Communication

How people understand and make sense of different risks varies based on several factors including education, income, gender, and ethnicity [23]. A key component to risk perception is how risks are communicated within civil society. Government agencies have increasingly recognized their responsibility to improve risk communication practice [24], [25], [26] and be sensitive to problems with ‘one-size fits all’ messaging [27]. Effective communication about risks depends as much on how well the risks themselves are understood (by both the sender and receiver of the message), the level of ‘trust’ in those responsible for managing the risk, and how confident people are in the information provided. For example, these factors can influence whether individuals will adopt messages about protective measures during an influenza pandemic [28], [29].

Globally, public health professionals and governments had long been expecting an influenza pandemic, investing heavily in preparedness plans. Many preparedness activities were geared towards an avian influenza pandemic (H5N1), including planning for the kind of communication messaging that would be needed. Early evaluations of different types of avian influenza messaging with health care providers and the general public suggested that risk information needs to be ‘just in time’ [30] and help to address confusion in terminology (i.e. how pandemic influenza is different from seasonal influenza) [31]. Going beyond mere publicity, open discussions and more targeted engagement was recommended for more controversial government or public health responses to the management of an influenza pandemic (i.e., social distancing measures, expediting drugs/vaccines, setting priorities for accessing limited supplies of drugs/vaccines) [32].

During a crisis like a pandemic influenza when information changes rapidly, having trusted and credible decision makers and communicators in place plays a pivotal role in the public uptake of risk communication messages [33], [34]. Risk communication messages must also be culturally appropriate [35], [36], [37], . For example, strategies involving Australian Aboriginal and Torres Strait Islander communities [22], [40], [41], Pacific Peoples, and Māori in New Zealand [21] were central to develop early engagement and partnerships to address delivery of culturally appropriate risk communication. Evaluations of these efforts concluded that more community based information dissemination mechanisms were needed [22], [40], [41], to avoid problems created by ‘one size’ pandemic warning strategies [21].

In Canada, the Public Health Agency of Canada (PHAC) played the lead role in managing the pandemic H1N1 response, although all aspects related to First Nations and Inuit (but not Metis) were directed by Health Canada [42]. These agencies, along with provincial counterparts like Manitoba Health, launched a series of risk communication campaigns designed to provide Canadians with consistent public health messages about the ways in which they could protect themselves and their families from contracting H1N1 influenza. Typical messaging included: wash hands frequently with soap and water or use a hand sanitizer; cover coughs and sneezes; stay home when sick; and get vaccinated. This messaging remains the same today for seasonal influenza [43], as well as for messages targeted specifically for First Nations and Inuit people [44]. While efforts were made to provide targeted communications to the Aboriginal population (i.e., through the Aboriginal People’s Television Network, translated radio announcements in local dialects, and a special information campaign through the Manitoba Metis Federation to its citizen membership), the impact of these efforts is unclear. To date, evaluations of pandemic H1N1 in Canada specific to Aboriginal people has concentrated on access and delivery issues in one remote area [45], case rates [16], [17], and how Aboriginal Canadians responded to the H1N1 vaccine [46].

One of the more unique aspects in the Manitoba pandemic response was the establishment of a federal/provincial/Aboriginal tri-partite table [47]. Aboriginal representation included members from First Nations and Metis self-government organizations. One goal of this tri-partite table was to ensure a prioritized distribution of the H1N1 vaccine, once it was available, to any citizen of First Nations (regardless of status/registry or location – either on or off reserve) or Metis heritage in the province.

Lastly, there was a pivotal moment in managing the response to pandemic H1N1 that was highly memorable in the minds of many Canadians. As per federal/provincial/territorial protocols, the province or territory sent pandemic supplies to a federal central repository for distribution to First Nations and Inuit within their geographic boundaries [47]. Though Manitoba Health sent its share of medical supplies as per the Canadian Pandemic Influenza Plan [48]for its First Nations residents, these were not received by some of the reserve communities in need. Those communities more severely affected by H1N1 during Wave 1 were frequently reported in the news as requesting supplies such as hand sanitizers and flu kits; one community even purchased its own supply out of frustration with the delays only to receive the federal shipment one week later [49]. To compound this problem, several body bags were sent as part of a regular federal resupply action to four Manitoba First Nations reserve communities. This shipment did not contain any protective measures like antivirals for the nursing station, hand sanitizers or flu kits for residents. This caused considerable political controversy and generated a great deal of media coverage. The First Nations Grand Chief for the area commented in the news that the shipment of body bags sent the message that the government was “writing off” the community; thus he made a further plea for H1N1 protective supplies [50]. Manitoba Health responded by sending a second set of supplies, this time directly to the affected communities [51], [52]. In this broader context, this article explores participant perspectives towards the management of pandemic H1N1.

## Methods

Focus group and key informant interview methods were used in this research. Focus groups were used for primary data collection with general public First Nations and Metis people. Key informant interviews were carried out with senior public health officials working within provincial or federal health offices, representatives from health systems organizations (e.g. a regional/local health authority), and from different First Nations and Metis self-government organizations with knowledge of H1N1 pandemic management and communications. Established protocols were followed in conducting focus groups [53], [54], [55] and key informant interviews [56], [57], [58], including having a skilled moderator and a second researcher present to record observational notes and audio-record conversations.

The Aboriginal communities that were the focus of this research played key roles in collaborating on identifying the community priorities that guided the research, on designing aspects of the study, and on facilitating the actual research (particularly in helping with recruitment activities for the focus groups). The research team included the Director of the Health & Wellness Department of the Manitoba Metis Federation as one the co-investigators, and First Nations community collaborators took the lead in initiating their involvement in the study.

Research ethics approval was obtained from the University of Manitoba Health Research Ethics Board (Reference numbers: H:2009∶258-pilot study; H2010∶008-national study). Interpreters were available if participants spoke French or the Metis language of Michif, however focus groups were conducted in English as all participants spoke English fluently. Participants were given the opportunity to provide either oral or written consent. The consent process for focus groups was audio-recorded, and people stated their name for the recording as well as their consent in lieu of signing a form. Much like producing written consent forms, this provides an audit trail of the consent process should the Research Ethics Board wish to verify process. This consent process was also approved by the Research Ethics Board and was done out of respect for people of First Nations, Inuit, and Metis heritage who feel, due to historical colonial injustices, that the signing of documents equates to the loss of rights and autonomy. As per protocols of working with the Manitoba Metis Federation and the remote First Nations community that was visited, additional ethical approval outside of a certified board was not required.

**Figure 1:**
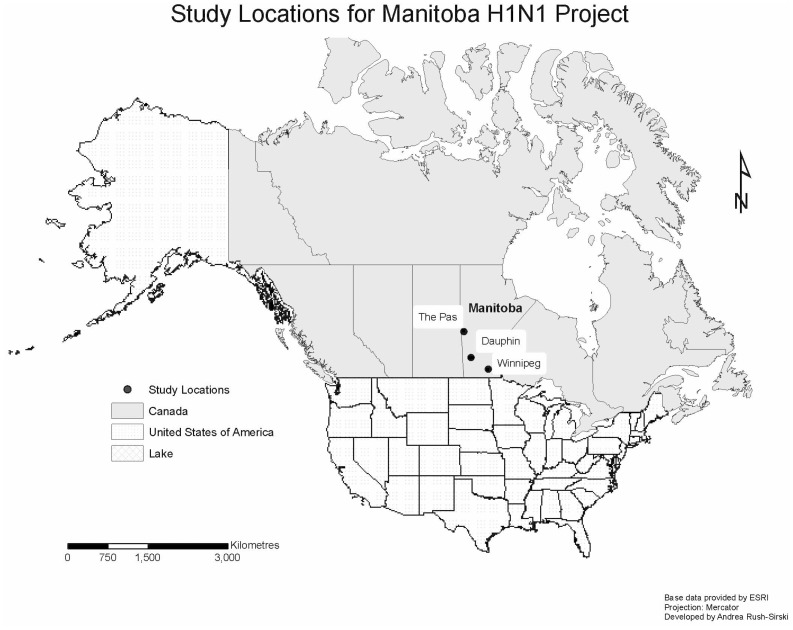
Study Locations for the Manitoba H1N1 Project.

A total of 23 focus groups were conducted with First Nations and Metis in Manitoba. Following Wave 1, at the invitation of the First Nations Chief, two focus groups of men and women were conducted in August 2009 with a northern Manitoba First Nations community that was severely affected by H1N1 influenza. Participants were recruited through the community Band Office. Following Wave 2 of the pandemic, an additional six focus groups were conducted in Winnipeg (the provincial capital city) in May and September 2010 with people who self-identified as urban Aboriginal (participants were not required to make specific declarations as First Nations, Metis, or both). Participants for this research were recruited through posters placed in locations commonly frequented by participants (e.g., Manitoba Metis Federation local offices, employment services, family resource centres, urban friendship centres, women’s resource centres, banking establishments, grocery stores and schools). Manitoba Metis Federation community collaborators also assisted in recruitment by using word-of-mouth notification for potential participants who may have lower levels of literacy. These eight focus groups were part of a pilot testing phase of both the focus group interview guide and a survey research instrument that was going to be used in a larger First Nations, Inuit and Metis project involving three different case studies, of which only the Metis case study would focus on H1N1. They involved men and women between the ages of 18–34, because this was believed to be the age group most susceptible to more serious H1N1 influenza outcomes. The remaining 15 focus groups were conducted with Metis men and women, stratified by age (18–34 years of age, 35–54 years of age, and 55 and older), in mid-province rural Manitoba and Winnipeg. On the recommendation of Manitoba Metis Federation collaborators, participants from the rural Metis focus groups were drawn from larger communities in order to recruit sufficient numbers. Urban Metis in Winnipeg were recruited using a similar strategy to that described for the urban Aboriginal focus groups. Despite efforts to recruit men, more women chose to participate in the research. Figure 1 shows a map of the Manitoba study area. The three nearest urban centres from which participants were drawn for the data presented in this paper are indicated. In deference to participant requests, the specific rural and remote towns from which participants came are not named here.

Participants were asked a series of questions related to health risks they faced in their community, responsibility for health (probing from individual level to level of the influence that health policies might mean for them), what they remembered of pandemic H1N1, the kinds of information they heard or may have sought, who they trusted to provide them with reliable information about H1N1, how they evaluated the information they received and so forth. Each participant received an honorarium of $50. All focus group conversations were audio-recorded, transcribed verbatim and verified for accuracy against the audio-file for analysis in NVivo9™.

A detailed codebook was developed from iterative engagement with a series of transcripts by the research team until all aspects of the data were being captured in the initial descriptive level of analysis. Three research assistants trained in NVivo9™demonstrated high agreement (inter-rater reliability scores of 94% for surface coding). Moreover, coders worked within the same physical space (often at the same time) and were able to resolve uncertain coding decisions through open discussion and consensus agreement. To identify broader themes in the dataset, a constant comparative and concept-development approach [59] was used. The project lead and a senior research assistant discussed the findings more broadly by critically reading content categories (i.e., nodes related to ‘trust’, ‘vaccine’, ‘risk’, ‘uptake of messaging’) to examine the subtext related to the themes presented in this article. These discussions were refined and nuanced following return of results to the communities from which we drew participants and through peer debriefing with the research team [60]. For example, researchers presented preliminary findings to the Manitoba Metis Federation’s Annual General Assembly in September 2011. In fact, some participants made a point of returning back to the conference booth after reviewing the materials and expressed their appreciation for sharing back the results and for capturing the issues that were of importance to them. More details about this process are available in a supplementary methods document outlining the larger project and specific strategies used [61].

Key informant interviews (n = 20) were conducted between March 2011 and June 2012. Participants were identified based on the key professional roles they played during the planning and response phases of pandemic H1N1. Honoraria were not provided. Key informant interview data were analyzed in a similar fashion to focus group data described above. While these interviews are not the main focus of this article, aspects of how H1N1 was managed and perceptions that health officials raised during these discussions are used to better contextualize the perspectives expressed by the First Nations and Metis focus group participants.

## Results and Discussion

A total of 193 people participated in the focus group discussions; 114 of these were women, 70 were men, and 9 did not indicate gender on the demographic sheet. Table 1 provides an overview of participant socio-economic and demographic characteristics. Overall, a disproportionate percentage of participants had not completed high school and many subsisted with low household incomes.

**Table 1:** Demographic/Socioeconomic Characteristics of Focus Group Participants vs MB Total Population.

	# of Participants	%	MB Total^3,4^	%
**Gender**				
Male	70	36.3	594,550	49
Female	114	59.1	613,715	51
Missing^1^	9	4.7		
**Age**				
18 to 34^2^	96	49.7	270,520	22
35 to 54	37	19.2	333,405	28
55 and older	47	24.4	321,115	27
Missing^1^	13	6.7		
**Marital Status**				
single	76	39.4	307,505	25
married/common law	65	33.7	529,595	44
Divorced/separated/widowed	17	8.8	152,630	13
Missing^1^	35	18.1		
**Education**				
Less than Grade 5	13	6.7	n/a	
Grades 5 to 10	67	34.7	n/a	
Grades 11 to 12	57	29.5	242,200^5^	20
Some/completeduniversity or college	20	10.4	309,940^5^	26
Missing^1^	36	18.7		
**Household Income**				
Less than $10,000	65	33.7	24,290	5
$10,001–$20,000	34	17.6	51,875	12
$20,001–$30,000	23	11.9	53,865	12
$30,001–$40,000	12	6.2	55,450	12
More than $40,000	11	5.7	263,290	59
Missing^1^	48	24.9		

1 Information not completed by participants at their own discretion. Marital Status, Education and Household Income were unfortunately not collected during first pilot testing of the instrument in the rural First Nations community prior to Wave 2 of H1N1. This represents 16 participants each for these three Missing values.

2 We over-sampled the 18–34 age category during our pilot testing phase (8 focus groups in total) as this was believed to be the age category at highest risk of more severe outcomes from pandemic H1N1.

3 Statistics Canada, 2011 Census of Population. At the time of writing, only age and sex data from the 2011 Census were available from Statistics Canada.

4 Statistics Canada, 2006 Census of Population. Includes all data on marital status, education, household income.

5 Statistics Canada, 2006 Census of Population. For MB Totals in education for Grades 11 to 12, number given is the High School Certificate or equivalent. For category of Some or completed university or college, number represents aggregate of those with College, CEGEP or other non-university certificate or diploma, university certificate or diploma below bachelor level, and university certificate, diploma or degree.

A sense of general stigmatization resulting from government action and public health messaging strongly emerged as participants discussed at length how they felt about the management of pandemic H1N1, articulated through three related aspects: 1) feelings that ’Aboriginal’ lives are less valued; 2) ‘Aboriginality’ as a risk factor; and 3) that there is a generalized ‘Aboriginal’ identity perpetuating a racialized ‘other’. In the presentation of results below, representative quotes will be showcased with the recognition that many similar statements were made by people across the entire data set.

### ‘Aboriginal’ Lives are Less Valued

General details about the ‘body bag’ incident as part of a resupply shipment have already been provided above. Although it was experienced by a small number of First Nations northern reserve communities, the incident served as a defining moment for how the management of H1N1 for Aboriginal people was perceived by focus group participants. Regardless of location (urban or rural/remote) or cultural and ethnic identity (First Nations, Metis, or both), there was a sense that ‘Aboriginal’ lives did not matter. While there were never any direct questions to participants related to the body bag incident, this memory resonated so strongly that it was raised without prompting.

“…they’re [the government] sending body bags and they’re not going to be helping us. They were really scaring me. ” Female, Rural Metis, 35–54.

“And about the racial aspect of it …when the government sent body bags instead of sending…the shots and vaccinations. They sent body bags.” Male, Urban Metis, 18–34.

“I couldn’t believe they did that [sent body bags], that was just the most tackiest shit the Government or any authorities have ever done.” Female, Urban Aboriginal, 18–34.

Concern over both access to, and legitimacy of, health services has been, and continues to be a real issue for many First Nations, Inuit and Metis. This was seen during pandemic H1N1 as fears mounted, especially in remote communities. Many of the supplies commonly referred to in health messaging for prevention and treatment (e.g., hand sanitizer, over the counter pain/fever medications) were not readily available in more isolated settings (i.e., no pharmacies or convenience stores exist). In this broader context, perceptions of First Nations and Metis being more ‘at risk’ or vulnerable to serious health consequences from pandemic H1N1 and being made a priority group for receiving the initial doses of the vaccine should be considered.

### The ‘Aboriginal’ Risk Factor

No genetic predisposition made First Nations and Metis people susceptible or sensitive to more severe outcomes of H1N1, but being ‘Aboriginal’ was frequently communicated as a risk factor. This communication created a great deal of confusion and distrust:

“Can I ask you a question? I don’t know if you can answer it but why…, who picks who is at the top of the list for the H1N1, like what were the studies? Why were Aboriginal people put on the top?” (Male, Urban Aboriginal, 18–34).

The air-lifting of many remotely located First Nations people in Manitoba during Wave 1 to hospitals in Winnipeg for treatment perpetuated the perception of being singled out in risk messaging. Beyond the confusion caused by this type of Aboriginal “at-risk” related communication, First Nations people faced challenging perceptions held by federal civil servants in Ottawa (Canada’s capital city), who seemed disconnected from the realities of many remote communities. When discussing some of the challenges faced in the Manitoba context, one provincial key informant expressed this frustration:

“Many of us had conversations with federal government employees who refused to believe that there were communities that didn’t have running water here and thought we were - quote – “making it up to make their minister look bad”. It was like, hello, these are your communities, and there is no running water…” (Provincial Key Informant).

Many First Nations and Metis remote communities have unmet housing needs, including the prevalent lack of running water. Until a vaccine was available, frequent hand washing was communicated as the most important protective behaviour an individual could adopt – an action made more difficult without running water. Some public health officials during interview conversations commented that the focus on pandemic H1N1 was on the ‘wrong’ pandemic: rather, the greater public health issue was and remains the social and economic circumstances that make some communities more vulnerable to negative health outcomes compared to the general population. From the perspectives of participants in this research, many of these broader circumstances are never systematically addressed.

### The Generalized ‘Aboriginal’ as the Racialized ‘Other’

The unexpected limited vaccine availability due to production delays contributed to a national public health response which established a list of priority groups to be ‘first in line’ to receive the vaccine. Nationally, the vaccine priority sequencing generically included persons residing in remote or isolated settings [62]. Provincially, Manitoba Health, supported by the tri-partite table, specifically listed ‘anyone of Aboriginal ancestry’ in its list for vaccine priority receipt [63]. Focus group participants felt that the language used to describe priority groups for ‘at risk’ populations were highly discriminatory:

“J: I was concerned because it seemed like they were picking like Aboriginals and Metis and the white people couldn’t even get [the] vaccine until these guys were getting it.

M: Almost like we were guinea pigs.

J: Ya, that was my concern.” Male, Rural Metis, 55+

“And how come they kind of singled out Aboriginal people? Is it like, they said that because they all live in crowded places, that's on the reserves but in, like in Winnipeg here so do Chinese people, they all live in one house. *[…]* it makes you feel kind of dirty *[…]* like oh Aboriginals we’re right on the top of the list, I mean there’s a lot of other different nationalities that live in packed houses in the city yet.” Female, Urban Aboriginal, 18–34.

This underlying sense of feeling like ‘guinea pigs’ was strong within the focus groups; some even adopted an ‘Aboriginal conspiracy’ sentiment:

*Female 1*: Tell me something, why would they give people shots here in the city, native people shots in the city and why they go drop two thousand body bags at a reserve.

*Male 1*: Because we’re native, no offense but because we’re native we’re lower class people to the government, we’re just expendable people…

*Male 2*: Well we’re just basically fodder, fodder for the cattle. (Urban Aboriginal, 18–34).

“I think H1N1 was man-made and I believe that they wanted to give us that vaccination to kill off the Native people.” (Female, Rural Metis, 18–65).

This is not to say that First Nations or Metis people were reluctant to get the H1N1 vaccine when it was made available. Vaccination rates in the province of Manitoba were 37% overall, whereas approximately 60% of the First Nations population chose to be immunized [47]. Rather, it shows how a generalized ‘Aboriginal’ identity serves to further racialize a group of people as a ‘problem’ in need of a solution. A key informant was not surprised by the ‘conspiracy theory’ reactions of participants. The motivations of tri-partite members (federal, provincial, First Nations and Metis self-government representatives) to prioritize Aboriginal people to receive the vaccine first reflected the continued unaddressed reality that Aboriginal people have a much lower health status compared to the general population:

“Well it was really interesting to me because we thought that the communication had been pretty good. I think we underestimated that feeling or that sense of “we’re always last so why are we first this time, like what’s going on here?” There was a complete lack of trust that the motivation was for the good of the people. And I mean if you look historically First Nations and Metis had never been prioritized for anything so why now? So if you put it in the context of history and then look at the fact that there was lots of communication on TV and radio and other places about the fact that they had abbreviated the safety testing of this vaccine to make it available and all the rest of it, I guess it’s not a bad assumption.” (Provincial Key Informant).

Several reasons could explain why First Nations and Metis participants felt stigmatized when made a ‘priority group’. When testing different risk communication messaging in preparation for what was to be an avian pandemic, Janssen and colleagues (2006) found that participating members of the general public and health care workers felt that ‘priority groups’ suggested an elitism creating the appearance of a social justice issue. Rather, they recommended that public health risk communicators avoid the ‘priority group’ term entirely in favour of simply listing the groups that would be first in line to receive dugs (like antivirals) or vaccines, and state the reason for the decision. In other Indigenous contexts, local (or community identified) leaders were the communicator [21], [41], but in Manitoba, federal or provincial senior leads in the respective agencies responsible for handling pandemic response conveyed the risk messages about the H1N1 virus and vaccine priority setting. Lastly, unlike in one international context [22], most public health officials and risk communicators failed to appreciate the strength of the post-colonial discourse underlying health and social issues for First Nations and Metis people.

### Conclusion

The management of H1N1 from a risk communication perspective was fraught with confusion, and from the participants’ perspective, resulted in supporting and perpetuating feelings of discrimination and vulnerability developed historically through colonization and government policies. Because anyone of Aboriginal ancestry in Manitoba was on the priority list for the vaccine, obtaining quick access was not an issue. Confidence in vaccine safety and that people of Aboriginal ancestry were not being used as human ‘guinea pigs’ was a substantial concern for participants. Ongoing underlying structural policies that contribute to and perpetuate the substantial social and economic disparities of First Nations, Inuit and Metis citizens compared to general population Canadians need to be addressed. Though not a risk communication issue, such efforts could best help to defend those individuals who may be more vulnerable to future pandemic outbreaks.

Results in this study offer instructive lessons to risk communicators and public health decision-makers. Though risk messages were transmitted in different dialects (e.g., Cree, Ojibwa, Michif, etc) and by different formats (radio, television, print, on-line, community sessions), the Canadian public health pandemic risk communication strategy was essentially a ‘one-size’ campaign. The nature of the Canadian confederation, with its federal-provincial-territorial division of powers and responsibilities makes it ripe for risk communication quagmires. Short of massive constitutional changes, or more efforts to address multi-jurisdictional issues regarding the delivery of health and social services to remote First Nations, Inuit and Metis citizens, some pragmatic recommendations stem from this research.

First, many public health professionals, communicators, and First Nations and Metis representative key informants who participated in this research all felt that communication was better with pandemic H1N1. Nevertheless, the target audience of a ‘first in line’ priority sequencing for the vaccine did not know why they were prioritized. Although Manitoba ensured that its pandemic response included a tri-partite table involving First Nations, and then later, Metis representatives, more work still needs to be done to advance communications and response around pandemic and other major public health issues. Communicating about what is being done and why certain decisions are being made cannot be handled like a public relations exercise, as citizens are quick to see through such disingenuous activities. Rather, creating more opportunities for open exchange and community-based dialogue is needed. While this is not easily done during a pandemic, taking steps now to fill this gap is urgently required.

Second, individuals who are highly placed to either make public health recommendations about risk reduction or who actually deliver the associated risk messages need to ensure the messaging is positioned within a post-colonial context for First Nations, Inuit and Metis audiences. Decision makers strategizing the mass immunization campaign need to communicate reasons for prioritizing groups. Rather than labelling an entire population to be ‘at risk’ because they demonstrate some risk factors known to increase vulnerability, priority groups should be defined by specific attributes. Instead of stigmatizing an entire population as ‘different’ based on ethnicity, efforts should be made to address the socio-economic and other disparities that make some members of this population particularly vulnerable. How to effectively deliver this message still requires appropriate testing with relevant segments of the public audience.

Third, functional partnerships between those generating the risk recommendations and First Nations, Inuit and Metis representatives need to be built in a positive and collaborative way, to help identify appropriate mechanisms to communicate health risks. The Manitoba Metis Federation, for example, collaborated with Manitoba Health to provide communications messaging for Manitoba Metis, the outcome of which will be the focus of a different article. Significantly, those in positions of leadership within First Nations, Inuit and Metis self-governing authorities are not always the same trusted spokespeople at the community level. While some attempts were made during pandemic H1N1 to incorporate ‘community-level’ voices, there are still significant opportunities for improvement. The challenge remains in continuing to build these relationships inter-pandemically.
